# A Functional SMAD2/3 Binding Site in the *PEX11*β Promoter Identifies a Role for TGFβ in Peroxisome Proliferation in Humans

**DOI:** 10.3389/fcell.2020.577637

**Published:** 2020-10-23

**Authors:** Afsoon S. Azadi, Ruth E. Carmichael, Werner J. Kovacs, Janet Koster, Suzan Kors, Hans R. Waterham, Michael Schrader

**Affiliations:** ^1^Biosciences, College of Life and Environmental Sciences, University of Exeter, Exeter, United Kingdom; ^2^Institute of Molecular Health Sciences, Swiss Federal Institute of Technology in Zürich (ETH Zürich), Zurich, Switzerland; ^3^Laboratory Genetic Metabolic Diseases, Amsterdam University Medical Centres, University of Amsterdam, Amsterdam, Netherlands

**Keywords:** peroxisomes, organelle dynamics, transcriptional regulation, peroxin, PEX11, transforming growth factor beta

## Abstract

In mammals, peroxisomes perform crucial functions in cellular metabolism, signaling and viral defense which are essential to the viability of the organism. Molecular cues triggered by changes in the cellular environment induce a dynamic response in peroxisomes, which manifests itself as a change in peroxisome number, altered enzyme levels and adaptations to the peroxisomal morphology. How the regulation of this process is integrated into the cell’s response to different stimuli, including the signaling pathways and factors involved, remains unclear. Here, a cell-based peroxisome proliferation assay has been applied to investigate the ability of different stimuli to induce peroxisome proliferation. We determined that serum stimulation, long-chain fatty acid supplementation and TGFβ application all increase peroxisome elongation, a prerequisite for proliferation. Time-resolved mRNA expression during the peroxisome proliferation cycle revealed a number of peroxins whose expression correlated with peroxisome elongation, including the β isoform of PEX11, but not the α or γ isoforms. An initial map of putative regulatory motif sites in the respective promoters showed a difference between binding sites in PEX11α and PEX11β, suggesting that these genes may be regulated by distinct pathways. A functional SMAD2/3 binding site in PEX11β points to the involvement of the TGFβ signaling pathway in expression of this gene and thus peroxisome proliferation/dynamics in humans.

## Introduction

Peroxisomes represent crucial subcellular compartments that are essential for human life and health. They perform key roles in cellular lipid metabolism, for example the breakdown and detoxification of fatty acids by α- and β-oxidation, and the synthesis of ether-phospholipids (e.g., plasmalogens, which are enriched in myelin sheaths), bile acids and polyunsaturated fatty acids (e.g., DHA) (Wanders, 2013a). Peroxisomes also contribute to hydrogen peroxide metabolism, cellular redox balance and redox signaling (Fransen and Lismont, 2018). Defects in peroxisome metabolic functions or biogenesis result in severe disorders with developmental and neurological defects (Wanders, 2013b; Braverman et al., 2016).

Peroxisomes are remarkably dynamic organelles, which respond to stimulation by adapting their morphology, abundance, and metabolic functions according to cellular needs. New peroxisomes can form from pre-existing peroxisomes by membrane growth and division, which results in their multiplication/proliferation (Schrader et al., 2016). Growth and division in mammalian cells follows a well-defined multi-step process of morphological alterations including elongation/remodeling of the peroxisomal membrane, constriction and recruitment of division factors (e.g., the adaptor proteins MFF, FIS1), and final membrane scission (by the dynamin-related GTPase DRP1) (Schrader et al., 2016). The membrane shaping peroxisomal membrane protein PEX11β contributes to multiple steps of peroxisomal growth and division including membrane deformation and elongation (Delille et al., 2010; Opaliński et al., 2011), recruitment of MFF and FIS1 to constriction sites (Koch et al., 2005; Koch and Brocard, 2012; Itoyama et al., 2013), and activation of DRP1 for membrane fission (Williams et al., 2015). Besides PEX11β, humans express two additional PEX11 isoforms, PEX11α and PEX11γ, but their exact functions are unclear and may differ, given that both isoforms cannot or only partially complement loss of PEX11β (Ebberink et al., 2012). So far, only patients with a defect in the β isoform of PEX11 have been identified, who present with enlarged peroxisomes, congenital cataracts, progressive hearing loss, short stature and neurological abnormalities (Ebberink et al., 2012; Taylor et al., 2017; Tian et al., 2019).

Although our understanding of the mechanisms by which peroxisomes proliferate is increasing, we have limited knowledge of how peroxisome biogenesis and division/multiplication is regulated by stimuli. In plants, it has been suggested that peroxisome proliferation can be stimulated by light, due to an increase in *PEX11b* transcription regulated by the transcription factors HYH and FHA3 (Desai and Hu, 2008; Desai et al., 2017). However, relatively little is known about how extracellular signals feed into peroxisome biogenesis in mammals and especially in humans. The best characterized regulatory pathway in mammals is the peroxisome proliferator-activated receptor (PPAR)-dependent pathway (Kliewer et al., 1992; Schrader et al., 2012b). PPARs are a family of transcription factors which modulate transcription of target genes in response to a variety of structurally diverse ligands, including xenobiotic chemicals called peroxisome proliferators, and both natural and synthetic fatty acids (Rakhshandehroo et al., 2010). PPARα is expressed predominantly in the liver, heart and brown adipose tissue, and is a major activator of fatty acid oxidation pathways (la Cour Poulsen et al., 2012). PPARγ is most highly expressed in white and brown adipose tissue, and functions as a master regulator of adipogenesis as well as a potent modulator of whole-body lipid metabolism and insulin sensitivity (Tontonoz and Spiegelman, 2008; Dubois et al., 2017; Kersten and Stienstra, 2017). PPARβ/δ is the most poorly characterized isoform, but is ubiquitously expressed and is thought to be important in lipid and cholesterol metabolism (Grygiel-Górniak, 2014). Upon ligand binding, PPARs hetero-dimerise with their binding partner, the 9-*cis*-retinoic acid receptor, RXRα, and bind to specific *cis*-acting DNA response elements known as PPRE, to initiate gene transcription. ChIP-seq analysis in human hepatoblastoma HepG2 cells revealed PPARα-occupied PPREs in genes encoding for peroxisomal enzymes such as ACOX1 (van der Meer et al., 2010), suggesting that their expression is regulated by PPARα.

PPARα agonists can significantly increase peroxisome number and the levels of fatty acid β-oxidation enzymes (Lazarow and De Duve, 1976; Schrader et al., 2016), but whilst rodents usually respond with a strong peroxisome proliferation phenotype, the effect in humans is very mild (Lawrence et al., 2001; Islinger et al., 2010). Although there is evidence for a functional PPRE in the promoter region of the *PEX11*α gene in both mouse (Shimizu et al., 2004) and human (Rakhshandehroo et al., 2010), there is little information so far on transcriptional regulation of other *PEX* genes in mammals, and especially humans. For example, there is currently no evidence for up-regulation of human PEX11β, as a key factor in peroxisome proliferation, following stimulation with PPARα agonists. In line with this, there is growing evidence that PPAR-independent pathways exist which can be activated by extracellular signals such as ROS and growth factors (Schrader and Fahimi, 2006; Bagattin et al., 2010; Fransen et al., 2012). Although the involvement of a variety of regulatory pathways in controlling transcription of peroxisome proliferation related genes under different conditions has been suggested, it is currently unclear how up-regulation of peroxisomal genes is linked to up-regulation of peroxisome proliferation.

Several studies have implicated the growth factor TGFβ in the regulation of peroxisomes, and while it has been suggested to both increase (Schrader et al., 1998a) and decrease (Oruqaj et al., 2015) peroxisome biogenesis under different conditions, the direct targets involved in this process are unclear. The TGFβ family consists of multifunctional proteins that regulate a diverse range of processes during development and tissue homeostasis, such as cell proliferation, apoptosis, autophagy, inflammation, angiogenesis, and epithelial-to-mesenchymal transition (Kitisin et al., 2007; Nagaraj and Datta, 2010; Horbelt et al., 2012; Massagué, 2012). There are three known isoforms of TGFβ (TGFβ1, TGFβ2, and TGFβ3) expressed in mammalian tissues; they contain highly conserved regions but are different in several amino acid sequences. All of these isoforms function through the same receptor signaling pathways (Horbelt et al., 2012). TGFβ isoforms bind to receptors at the cell surface, and recruit two type I receptors and two type II receptors forming a tetrameric complex (Massagué, 2012). Activated TGFβ superfamily receptors induce a phosphorylation cascade, from receptor phosphorylation to subsequent phosphorylation and activation of downstream signal transducer R-SMAD transcription factors (receptor-activated SMADs, including SMAD2/3) (Hill, 2016; Miyazawa and Miyazono, 2017). Phosphorylated R-SMADs form a hetero-oligomeric (often trimeric) complex with SMAD4. This R-SMAD/SMAD4 complex is imported into the nucleus where it regulates the expression of target genes by direct binding to specific motifs in the target gene promoter and/or through interaction with transcriptional cofactors in a cell-type-specific manner (Hariharan and Pillai, 2008; Kamato et al., 2013). SMAD target genes include the transcription factors PGC-1α and PPARγ, classically thought to regulate peroxisome proliferation, with elevated TGFβ signaling leading to systemic insulin resistance and hepatic steatosis through decreased expression of these target proteins (Yadav et al., 2011; Sohn et al., 2012). Additionally, PPARα may act upstream of TGFβ-dependent gene transcription, with PPARα ligands reported to decrease TGFβ-induced integrin expression by inhibiting SMAD4 complex formation (Kintscher et al., 2002), suggesting a complex interplay between TGFβ signaling and PPAR function.

Since deficiencies in peroxisome proliferation have been associated with a variety of disease states, including liver diseases and neurological dysfunction, as well as cellular aging (Cimini et al., 2009; Titorenko and Terlecky, 2011; Schrader et al., 2014; Passmore et al., 2020), a clearer understanding of the mechanisms and signaling pathways that control peroxisome plasticity could allow for modulation of peroxisome abundance to improve cellular function in health and disease. In this study, we aimed to identify novel signaling pathways and associated factors involved in peroxisome dynamics in humans, using a cell-based assay to investigate different stimuli and their ability to induce peroxisome proliferation. Examination of mRNA levels during peroxisome proliferation suggested differential regulation of peroxisomal genes correlating with their cellular function. Specifically, we identified differential regulation and functions of the PEX11 isoforms PEX11α and PEX11β in peroxisome dynamics. A functional SMAD2/3 binding site in the promoter of PEX11β suggests a novel link between the canonical TGFβ signaling pathway and the induction of peroxisome proliferation, potentially via PEX11β expression, providing new insights into the regulation of peroxisome dynamics in humans.

## Materials and Methods

### Plasmids and Antibodies

For cloning of *PEX11*β promoter regions, the candidate promoter region (1,302 bp for the wild type and 1,296 bp for the mutant lacking the SMAD2/3 binding site) of human *PEX11*β was synthesized (Eurofins Genomics) and then cloned into the pGL3-basic vector upstream of the firefly luciferase coding sequence (#E1751, Promega). The pGL3-basic vector without a promoter was used as a negative control. pRL-TK vector (#E2231, Promega) was used as internal control vector to normalize luciferase activities. See Supplementary Table 1 for details of plasmids used in this study, Supplementary Table 2 for plasmids generated in this study and Supplementary Table 3 for details of qPCR primers used. All constructs produced were confirmed by sequencing (Eurofins Genomics). Details of all antibodies used in this study can be found in Supplementary Table 4.

### Cell Culture and Cell-Based Peroxisome Proliferation Assay

For routine culture, HepG2 cells (human hepatoblastoma cells, HB8065; ATCC) were maintained in Minimal Essential Medium (MEM; Life Technologies) supplemented with 10% FBS, (100 U/ml penicillin and 100 μg/ml streptomycin) at 37°C in a 5% CO_2_-humidified incubator. COS-7 (African green monkey kidney cells, CRL-1651; ATCC), PEX11β-deficient skin fibroblasts (Ebberink et al., 2012) and PEX11 KO HeLa cells were cultured in Dulbecco’s Modified Eagle’s Medium (DMEM; Life Technologies) supplemented as above. COS-7 cells were transfected using diethylaminoethyl (DEAE)-dextran (Sigma-Aldrich) as described (Bonekamp et al., 2010). For stimulation experiments, HepG2 cells were cultured in serum-free MEM with N1 supplement [Sigma: 0.5 mg/ml recombinant human insulin, 0.5 mg/ml human transferrin (partially iron-saturated), 0.5 μg/ml sodium selenite, 1.6 mg/ml putrescine, and 0.73 μg/ml progesterone] containing 0.25% BSA. For morphological analysis of peroxisomes, 70,000/cm^2^ HepG2 cells were seeded on collagen (Serva)-coated glass coverslips. Compounds/inhibitors were added 6 h after seeding and cells were processed for immunofluorescence at different time points after incubation (as indicated in Figures). Fatty acid stocks (Sigma-Aldrich) were dissolved in ethanol. WY-14643 (#C7081), Troglitazone (#T2573), GW9962 (#M6191), Rapamycin (#R0395), LY2109761 (#SML2051), and SB431542 (#616461) were obtained from Sigma-Aldrich. Recombinant human TGFβ was obtained from R&D Systems (#240-B). Stimulation with 10% FBS served as a positive control for peroxisome elongation/proliferation.

### Generation of PEX11-Deficient HeLa Cells by CRISPR-Cas9

For each *PEX11* gene, two different sgRNA guide sequences targeting an exon of *PEX11*α, *PEX11*β, or *PEX11*γ were selected using the CRISPR Design Tool^1^ (Ran et al., 2013) (Supplementary Table 5). Complementary guide oligos were annealed and subsequently cloned into the pX458 [-pSpCas9(BB)-2A-GFP] plasmid (Addgene). Cells were transfected in six-well plates with 2 μg plasmid containing sgRNA targeting *PEX11*α, *PEX11*β or *PEX11*γ (jetPRIME DNA Transfection). Single GFP-positive cells were sorted by fluorescence-activated cell sorting into 96-well plates (SH800 Cell Sorter, Sony Biotechnology) as described (Ran et al., 2013). After ∼4 weeks, DNA was isolated from multiple expanded single colonies, and the targeted exon of the *PEX11* genes were PCR amplified using Phire Hot Start II DNA Polymerase (Thermo Fisher Scientific) and subsequently Sanger sequenced to confirm clonal populations with heterozygous mutations in *PEX11*α, *PEX11*β, and *PEX11*γ (Supplementary Table 5).

### Transfection and Dual Luciferase Reporter Assay

2 × 10^5^ HepG2 cells/well were seeded the day before transfection in 6-well plates in 1.5 ml complete growth MEM. 1 μg of the desired pGL3 vector DNA and 40 ng pRL-TK vector DNA (ratio 25:1) was transfected per well using Lipofectamine 3000 (Invitrogen) according to instructions.

48 h after transfection, cells were seeded in MEM/N1 media in 96 cell plates. 6 h after seeding, cells were treated with TGFβ, FBS or left untreated and were incubated for 24 h prior to performing the Dual-Luciferase Reporter assay (Promega, #E1910) with a MicroLumat Plus LB 96V luminometer (Berthold Technologies), according to manufacturer’s instructions. Luminescence was measured for 10 s, and each reaction was measured three times. For analysis, Firefly luciferase activities were normalized to Renilla luminescence in each well.

### RNA Isolation and Quantitative PCR

Total RNA was prepared from HepG2 cells with the NucleoSpin RNA Kit (#740955.50; Macherey-Nagel) and DNA was removed by on-column digestion with rDNase. cDNA was synthesized from 2 μg RNA with random hexamer primers using the High-Capacity RNA-to-cDNA Kit (#4368813; Applied Biosystems) and diluted 10-fold in sterile H_2_0. The real-time qPCR reaction was performed on a Roche LightCycler LC480 instrument in duplicates using 10 ng cDNA, 7.5 pmol forward and reverse primers, and the 2× KAPA SYBR FAST qPCR Mastermix (#KK4601; KAPA Biosystems). Thermal cycling was carried out with a 5 min denaturation step at 95°C, followed by 45 three-step cycles: 10 s at 95°C, 10 s at 60°C, and 10 s at 72°C. Melt curve analysis was carried out to confirm the specific amplification of a target gene and absence of primer dimers. Relative mRNA amount was calculated using the comparative threshold cycle method. 18S rRNA was used as the invariant control.

### Immunofluorescence and Microscopy

Cells were processed for immunofluorescence 24–48 h after transfection. Cells grown on collagen-coated glass coverslips were fixed for 20 min with 4% (w/v) para-formaldehyde in PBS (pH 7.4), permeabilized with 0.2% (w/v) Triton X-100 for 10 min and blocked with 1% BSA for 10 min. Blocked cells were sequentially incubated with primary and secondary antibodies for 1 h in a humid chamber. Coverslips were washed with ddH_2_O to remove PBS and mounted with Mowiol medium (containing 25% n-propyl-gallate as anti-fading reagent) on glass slides. All immunofluorescence steps were performed at room temperature and cells were washed three times with PBS, pH 7.4 between each individual step. Cell imaging was performed using an Olympus IX81 microscope equipped with an UPlanSApo 100×/1.40 oil objective (Olympus). Digital images were taken with a CoolSNAP HQ2 CCD camera. Representative images only were adjusted for contrast and brightness using the Olympus Soft Imaging Viewer software (Olympus) and MetaMorph 7 (Molecular Devices).

### Promoter Analysis

For each *PEX11* isoform gene, regions 10kb upstream of the start codon were scanned base-pair by base-pair for regulatory elements using the GimmeMotif programme (van Heeringen and Veenstra, 2010). To obtain transcription factor binding site motifs to search for, position weight matrices representing the DNA binding preferences of 700 transcription factors were imported from the JASPAR CORE database^2^. *Bona fide* binding sites in the regulatory region for each chosen gene were defined as having a higher than 89% potential match to a JASPAR motif. Binding efficiency to SMAD2/3 motifs was calculated relative to the JASPAR position weight matrix consensus sequence (ID MA0513.1). In order to eliminate non-active motif binding sites based on chromosome structure, the presence of H3K4me3, H3K36me3, H3K27ac histone marks and absence of H3K27m3 near the potential motif was checked with the USCS Genome Browser as indicators of transcriptionally active sites in HepG2 cells. The ENCODE database of transcription factor binding tracks was used to exclude transcriptionally silent sites in each gene (those with a score of <500 out of 1000 for ChIP-seq evidence of transcription factor binding).

### Quantification and Statistical Analysis of Data

For quantitative analysis of peroxisome morphology, 100–200 cells per coverslip were examined and categorized possessing elongated/tubular (>2 μm in length) or spherical peroxisomes (0.3–2 μm; including rod-shaped peroxisomes) (Schrader et al., 1996). For quantification of peroxisome number, images were acquired in the focal plane of the nucleus, and peroxisome number per cell was determined using an in-house ImageJ macro (Schneider et al., 2012; Passmore et al., 2020) based on the ‘Analyze Particles’ function. Usually, three coverslips per preparation were analyzed, and three independent experiments were performed. Statistical analyses were performed using GraphPad Prism 5 software. Data are presented as means ± SD. Two-tailed unpaired *t*-tests (for experiments with two groups) or one-way ANOVAs with appropriate *post hoc* tests (for experiments with three or more groups) were used to determine statistical differences against control values (see Figure Legends for details). ^∗^*p* < 0.05, ^∗∗^*p* < 0.01, ^∗∗∗^*p* < 0.001, ns, non-significant.

## Results

### Validation of a Cell-Based Peroxisome Proliferation Assay

**FIGURE 1:**
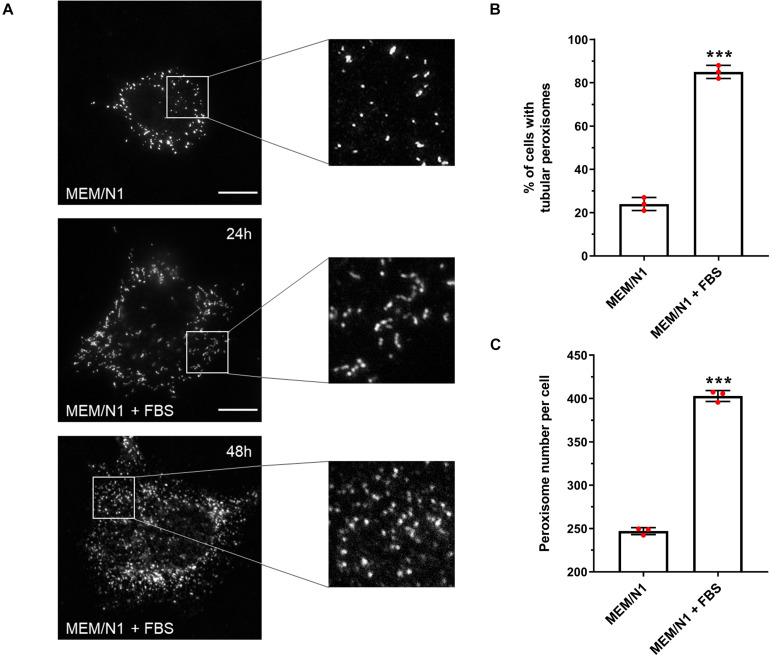
Effect of serum on peroxisome morphology/proliferation in HepG2 cells. **(A)** Representative images of cells cultured in MEM/N1 and cells stimulated with 10% FBS (MEM/N1 + FBS). Cells were processed for immunofluorescence after 24 or 48 h of stimulation and stained with anti-PEX14 antibodies. Bars, 10 μm **(B)** Quantitative analysis of peroxisome elongation after 24 h, expressed as percentage of cells exhibiting tubular peroxisomes. Data from 3 independent experiments (*n* = 300 cells in each condition); analyzed by two-tailed unpaired *t*-test. **(C)** Quantitative analysis of peroxisome number per cell after 48 h. Data from 3 independent experiments (*n* = 10 cells for each condition), analyzed by two-tailed unpaired *t*-test; ***p < 0.001.

Expansion of the peroxisomal membrane is the first in a sequence of morphological changes that occur during peroxisomal growth and division in mammalian cells (Schrader et al., 2016). In human hepatoblastoma HepG2 cells, elongated or tubular peroxisomes increase in number for approximately 24 h after seeding; the number of tubular peroxisomes decreases after this time point as fission occurs and most of the cells show spherical peroxisomal forms (Schrader et al., 1996). This membrane growth and subsequent fission contributes to the multiplication/proliferation of peroxisomes under conditions of cellular growth. In order to characterize and measure the progression of peroxisome proliferation in HepG2 cells, we have established a cell-based proliferation assay to measure synchronous peroxisome dynamics (Figure 1). Alterations in peroxisome morphology under different culture conditions were quantified over time by immunofluorescence microscopy, staining for the peroxisomal membrane protein PEX14 (Nguyen et al., 2006; Grant et al., 2013). Under standard culture conditions (MEM with 10% FBS [MEM/FBS]), the number of tubular peroxisomes increased in a time-dependent manner in HepG2 cells seeded at a defined density, as previously reported (Schrader et al., 1996). In cells cultured in defined serum-free media (MEM with N1 supplement [MEM/N1]), however, the majority of peroxisomes remained spherical after 24 h of culture (Figures 1A,B), creating a ‘basal’ state from which peroxisome tubulation could be induced and measured. By synchronizing peroxisomes in a spherical form in this way, a change in peroxisome morphology could be monitored to determine modulators of peroxisome proliferation.

**FIGURE 2:**
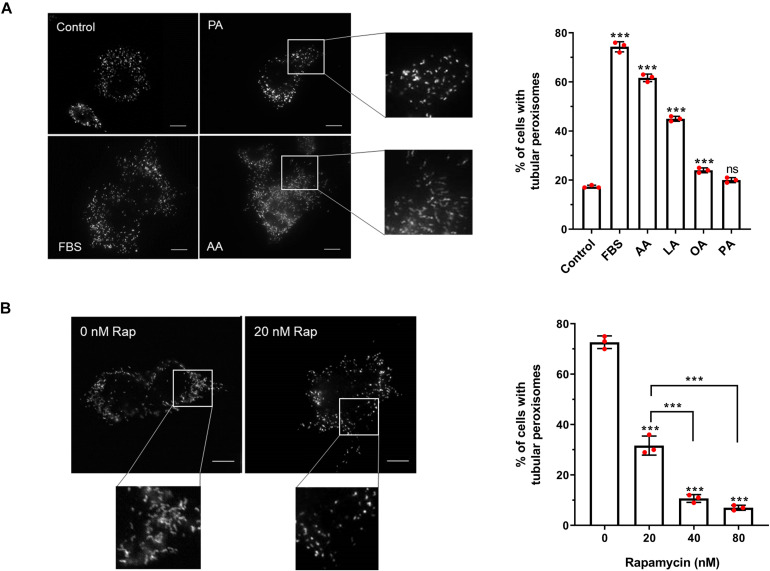
Effect of fatty acids and rapamycin on peroxisome elongation in HepG2 cells. **(A)** Quantitative analysis of peroxisome morphology upon stimulation with 50 μM fatty acids in HepG2 cells cultured in MEM/N1 for 24 h. Note the strong stimulatory effect of PUFAs (AA and LA). **(B)** Quantitative analysis of peroxisome morphology after treatment of HepG2 cells with rapamycin and subsequent serum stimulation. Data in **(A,B)** are based on immunofluorescence microscopy using anti-PEX14 from 3 independent experiments (*n* = 300 cells in each condition); analyzed by one-way ANOVA with Dunnett’s **(A)** or Tukey’s **(B)** *post hoc* test; ***p < 0.001. AA, arachidonic acid; LA, linoleic acid; OA, oleic acid; PA, palmitic acid.

To validate that our assay system could be used to detect and follow changes in peroxisome morphology in response to environmental stimuli, HepG2 cells were incubated for 6 h in MEM/N1, where cells display spherical and rod-shaped peroxisome morphologies, then exposed to 10% FBS for 24 h (‘serum stimulation’). Peroxisome morphology was again assessed by PEX14 immunofluorescence. Serum stimulation increased the proportion of cells with tubular peroxisomes to more than 80% (Figure 1B), whereas peroxisomes remained predominantly spherical in cells kept in serum-free medium. Furthermore, after 48 h, the number of peroxisomes per cell was 60% higher in serum-stimulated cells compared to those cultured in MEM/N1 (Figure 1C). This confirms that elongated peroxisomes, which peak in number after 24 h, divide by fission and contribute to peroxisome proliferation. These observations indicate that peroxisomal growth and division is reduced under serum-free culture conditions, and therefore that the peroxisomal compartment is responsive to serum stimulation.

To test if cellular growth in MEM/N1 is comparable to standard growth conditions, we determined and compared cellular growth over time. Cells cultured in MEM/N1 display slightly lower growth rates when compared to cells cultured in MEM/FBS (Supplementary Figure 1). However, the cells are actively proliferating and an exponential increase in cell density arises. It should, however, be noted that the cells appear smaller and are not as extensively spread as HepG2 cells grown under standard conditions.

With this cell-based assay, we have established an inducible system to dissect the distinct phases of peroxisome proliferation in a synchronous manner, and test different stimuli and their effect on peroxisome dynamics and proliferation in a quantitative fashion. To verify the assay, we first applied arachidonic acid (AA) [C20:4 (ω-6)] as a positive control. This PUFA serves as a precursor for the synthesis of a number of biologically active lipid mediators and has been reported to induce peroxisome elongation/proliferation in a previous study (Schrader et al., 1998a). Indeed, our results show that 50 μM AA causes a prominent increase in the percentage of cells with tubular peroxisomes (∼3.5-fold increase compared to the control) (Figure 4A), a similar magnitude of effect as when serum is added to the MEM/N1 medium. The addition of the PUFA linoleic acid (LA) [C18:2 (ω-6)] promoted peroxisome elongation, but less than AA or serum stimulation (∼2.6-fold increase compared to the control), while oleic acid (OA) [C18:1(ω-9)] also had a small but significant stimulatory effect on peroxisome elongation (∼1.4-fold increase compared to the control). However, the saturated fatty acid palmitic acid (PA) (C16:0) was not able to induce peroxisome membrane expansion at the concentrations applied (Figure 4A), even though, as observed for AA, LA, and OA, the number and size of lipid droplets was increased in the cells (not shown), indicating that all the fatty acids are efficiently taken up and stored by the cells. Together, this suggests a correlation between fatty acid chain length and/or degree of saturation and their ability to induce peroxisome elongation. Shorter-chain fatty acids are preferential substrates for mitochondrial β-oxidation, while the longer-chain fatty acids, which induce peroxisome elongation, can be β-oxidized in peroxisomes and/or are involved in the synthesis of cellular lipid mediators.

PUFAs such as AA and LA can act as PPAR ligands (Varga et al., 2011), so we next tested the ability of pharmacological PPAR ligands to induce peroxisome elongation in our experimental system. Interestingly, they did not induce a pronounced peroxisome elongation in our experimental system. While the PPARα agonist Wy-14,643 and the PPARγ agonist troglitazone did induce some peroxisome elongation at the higher concentrations tested (Supplementary Figure 2), this was a significantly smaller effect than the FBS-induced elongation, suggesting PPAR activation alone is not sufficient to account for the proliferative effect of serum on peroxisomes, which is in line with the mild effects described for peroxisome proliferators on human cells. Furthermore, the PPARγ antagonist GW9962, which inhibits PPARγ binding to its binding elements (Liu et al., 2016), caused a small decrease in peroxisome elongation compared to controls, but did not occlude the peroxisome elongation/proliferation induced by serum. This implies that serum contains factors which can induce peroxisome elongation/proliferation independently of PPARs.

Apart from fatty acids, the stimulatory effect of serum on peroxisome dynamics and proliferation may be due to the involvement of growth factor-dependent pathways, such as the mTOR pathway. In order to investigate the involvement of the mTOR pathway in peroxisome elongation/proliferation in our assay, cells cultured in MEM/N1 medium were pre-treated with the mTOR inhibitor rapamycin prior to serum stimulation. Two hours of rapamycin pre-treatment blocked peroxisome elongation in response to serum stimulation in a dose-dependent manner (Figure 4B), supporting our hypothesis that growth factor-mediated signaling pathways (specifically, the mTOR pathway) may play a role in the initiation of peroxisome proliferation during cellular growth.

### Differential mRNA Profiles During Peroxisome Proliferation

**FIGURE 3:**
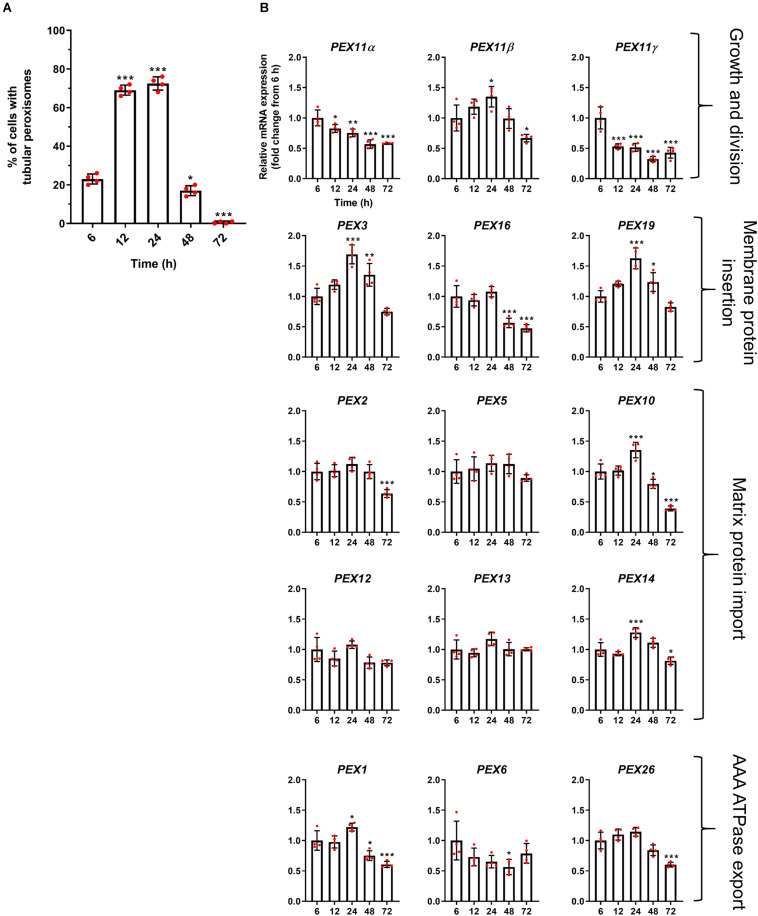
Correlation of peroxisome morphology and mRNA abundance profiles of PEX genes in HepG2 cells during a time course. **(A)** Quantitative analysis of tubular peroxisomes in HepG2 cells monitored over time in MEM/FBS and processed for immunofluorescence. Data from 4 independent experiments (*n* = 300 cells in each condition); analyzed by one-way ANOVA with Dunnett’s *post hoc* test. **(B)** qPCR analysis of relative mRNA levels of a set of *PEX* genes normalized to 18S rRNA expression over time. Note that *PEX11*β mRNA levels correlate with the proportion of cells showing tubular peroxisome morphology, emphasizing its role in peroxisome elongation and division. In contrast, *PEX11*α mRNA levels do not correlate with changes in peroxisome morphology. Data from 4 independent experiments; analyzed by one-way ANOVA with Dunnett’s *post hoc* test; *p < 0.05, **p < 0.01, ***p < 0.001.

Little is known about the regulation of peroxins and peroxisomal membrane proteins and the relationship between gene expression and peroxisome proliferation, in particular in humans. The only peroxin whose expression has so far been linked to peroxisome proliferation in humans is PEX11 (Schrader et al., 2016). We therefore set out to profile which peroxisomal genes may be involved in peroxisomal growth and division by correlating their time-resolved mRNA expression profiles with distinct peroxisome proliferation events in our synchronized cell model. mRNA was extracted at given time points after serum-stimulated induction of peroxisome proliferation, corresponding to distinct phases in the elongation and division process (Schrader et al., 1998a) (Figure 3A) – at 6 h, when peroxisome elongation begins; at 12 and 24 h when the number of elongated peroxisomes increases and peaks; and at 48 and 72 h, when division of the elongated peroxisomes has occurred, and they return to their spherical shape. qPCR was used to quantify changes in relative mRNA levels of a number of candidate peroxisomal genes across these stages of proliferation, normalized to 18S rRNA levels.

**FIGURE 4:**
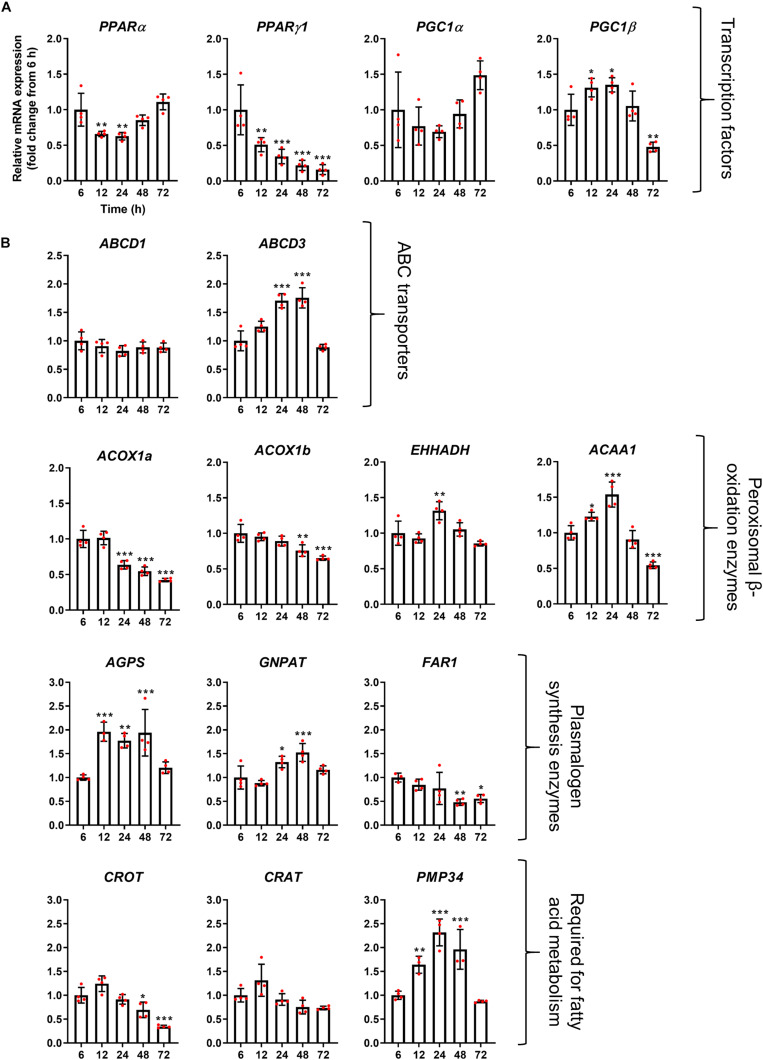
mRNA abundance profiles of peroxisomal transcription factors/co-factors **(A)** and peroxisomal genes associated with fatty acid beta-oxidation **(B)** in HepG2 cells during a time course. qPCR analysis of relative mRNA levels of the indicated genes, normalized to 18S rRNA expression over time. Data from 4 independent experiments; analyzed by one-way ANOVA with Dunnett’s *post hoc* test; *p < 0.05, **p < 0.01, ***p < 0.001.

We first investigated the mRNA profiles of the three mammalian isoforms of *PEX11* (α, β, and γ) during peroxisome proliferation, as these proteins are thought to be involved in the peroxisome growth and division cycle (Figure 3B). Interestingly, while *PEX11*β mRNA levels correlated well with the peroxisome proliferation cycle, *PEX11*α and *PEX11*γ did not, with their mRNA levels declining over time. These findings support the view that *PEX11*β is the key PEX11 isoform promoting peroxisome elongation and subsequent division, with *PEX11*α and *PEX11*γ playing more subtle or different roles.

Interestingly, mRNA levels of the early peroxins *PEX3* and *PEX19* were also observed to correlate with the peroxisome proliferation cycle, whereas *PEX16* remained unchanged and declined at later time points (Figure 3B). As these early peroxins are required for the insertion of peroxisomal membrane proteins including PEX11β, increased mRNA levels of those early peroxins is consistent with a requirement for PEX11β in proliferation. Other peroxins such as *PEX2*, *PEX5*, *PEX10*, *PEX12*, *PEX13*, and *PEX14*, which are involved in peroxisomal matrix protein import, showed a variety of expression profiles: a minor increase in mRNA expression after 24 h followed by a decline (*PEX10* and *PEX14*), a slight decline after 72 h (*PEX2*) or no change over the time course (*PEX5*, *PEX12*, and *PEX13*) (Figure 3B). Similarly, mRNA levels of the AAA-ATPase export complex components (*PEX1*, *PEX6*, and *PEX26*) did not correlate with the peroxisome proliferation cycle either, showing a minor increase after 24 h followed by a decline (*PEX1*), a decrease in expression after 48 h which recovers by 72 h (*PEX6*) and a slight decline after 72 h (*PEX26*), respectively.

**FIGURE 5:**
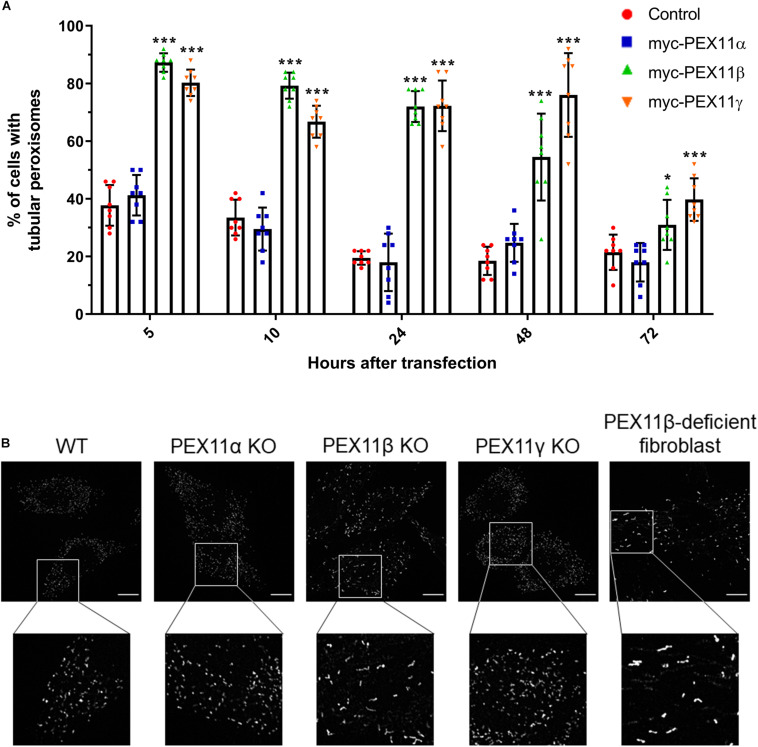
The human PEX11 isoforms affect peroxisome morphology differently. **(A)** Quantitative analysis of peroxisome morphologies in cells expressing different human PEX11 isoforms. COS-7 cells were transfected with Myc-PEX11α, Myc-PEX11β, or Myc-PEX11γ, processed for immunofluorescence using anti-Myc and anti-PEX14 antibodies at the indicated time points after transfection, and analyzed by microscopy. Note the prominent effect of Myc-PEX11β and Myc-PEX11γ on peroxisome elongation/division, in contrast to Myc-PEX11α expression, which is similar to mock-transfected controls (Con). Data from 4 independent experiments; analyzed by one-way ANOVA with Dunnett’s *post hoc* test for each time point; *p < 0.05, ***p < 0.001. **(B)** HeLa PEX11 KO cells and controls, and PEX11β-deficient fibroblasts were processed for immunofluorescence microscopy using antibodies against PEX14. Note the reduced number and elongation of peroxisomes in the PEX11β-deficient cells. Bars, 10 μm.

The mRNA profile of the transcription factors *PPAR*α, *PPAR*γ*1*, and *PGC1*α, which are classically thought to be involved in the upregulation of peroxisomal matrix protein expression (in particular β-oxidation enzymes in rodents), did not correlate with peroxisomal morphology alterations, and expression of those transcription factors rather declined over time, or remained constant (*PGC1*α) (Figure 5A). These findings may indicate that the observed morphological alterations are independent of those transcription factors. It has, however, been reported that PPARα expression is upregulated in HepG2 cells at the mRNA and protein level following prolonged incubation times in culture (Stier et al., 1998). Notably, mRNA levels of the transcription factor *PGC1*β does seem to correlate with the peroxisome proliferation cycle, suggesting this could play a role in peroxisome elongation and/or division. Interestingly, mRNA levels of the peroxisomal ABC transporter *ABCD3* (*PMP70*) also followed the morphological changes of peroxisomes, whereas mRNA levels for *ABCD1*, the ABC transporter for VLCFA, remained unchanged (Figure 5B). mRNA levels of *ACOX1a* and *ACOX1b*, key enzymes in the first step of peroxisomal fatty acid β-oxidation, was slightly reduced over time, while mRNA levels of *EHHADH*, which catalyzes the 2nd and 3rd step of peroxisomal β-oxidation, were upregulated after 24 h, and expression of *ACAA1*, which catalyzes the final step, was increased after 12 and 24 h but decreased after 72 h (Figure 5B). mRNA levels for other proteins required for peroxisomal β-oxidation, such as carnitine metabolism (*CROT* and *CRAT*), also follow different patterns over time, whereas the peroxisomal membrane protein *PMP34* (cofactor transport) correlated with the peroxisome proliferation cycle. Furthermore, mRNA levels of peroxisomal enzymes involved in plasmalogen synthesis showed distinct profiles, either increasing (*AGPS* and *GNPAT*) or decreasing (*FAR1*) at later time points (Figure 5B). Together this suggests that, in some cases, peroxisomal genes that perform similar functions (e.g., enzymes involved in β-oxidation or plasmalogen synthesis) may be independently regulated during proliferation.

### The Mammalian PEX11 Isoforms Differ in Their Membrane Elongation-Inducing Properties

**FIGURE 6:**
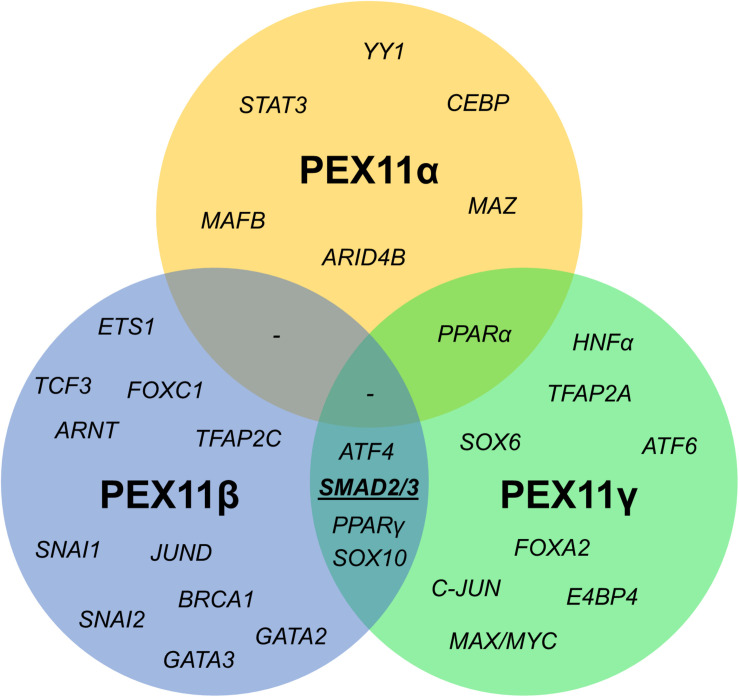
Venn diagram of shared transcription factor binding sites within the promoter regions of human *PEX11* isoforms. Potential binding sites for SMAD2/3 (activated by the TGFβ pathway) in *PEX11*γ and *PEX11*β were predicted to be located in a transcriptionally active area according to histone marks.

Our results from the mRNA profiling during peroxisome proliferation suggested that the three human PEX11 isoforms may play different roles in the regulation of peroxisome dynamics. In order to compare the functional effects of these isoforms on peroxisome morphology, COS-7 cells cultured in serum-containing media (to promote peroxisome growth and division) were transfected with Myc-PEX11α, Myc-PEX11β, or Myc-PEX11γ, processed for immunofluorescence after 5–72 h using anti-Myc and anti-PEX14 antibodies, and analyzed (Figure 6A). Quantification of peroxisome morphology revealed that overexpression of both Myc-PEX11β and Myc-PEX11γ had already induced the formation of tubular peroxisomes just 5 h after transfection. The number of cells with tubular peroxisomes declined over time, likely due to division of elongated peroxisomes into spherical organelles. In contrast, expression of Myc-PEX11α did not induce peroxisome elongation, and values were similar to controls confirming previous studies in both HepG2 and COS-7 cells (Schrader et al., 1998b; Delille et al., 2010). These findings indicate that the PEX11 isoforms affect peroxisome morphology differently, with β and γ being capable of inducing peroxisome elongation, whereas α cannot.

We also analyzed peroxisome morphology in HeLa CRISPR knock out (KO) cells lacking PEX11α, PEX11β, or PEX11γ, cultured in serum-containing media. Immunofluorescence microscopy using anti-PEX14 antibodies revealed a spherical peroxisome morphology in PEX11α and PEX11γ KO cells, which was comparable to controls (Figure 6B). In contrast, peroxisomes in PEX11β KO cells were reduced in number and showed a rod-shaped, slightly elongated morphology (Figure 6B). A similar peroxisome morphology was observed in skin fibroblasts from patients with a loss of PEX11β (Figure 6B) (Ebberink et al., 2012). At first glance, the slightly elongated peroxisome morphology in PEX11β-deficient cells may contradict the proposed function of PEX11β as a driver of membrane elongation. However, PEX11β also plays a role in peroxisome division as it acts as a GTPase activating protein (GAP) on the fission GTPase DRP1 (Williams et al., 2015). We suggest that as a consequence of this GAP function, peroxisome division is reduced in the absence of PEX11β leading to a slight elongation of peroxisomes, perhaps mediated by PEX11γ, which also has membrane elongating properties (Figure 6A) and can partially complement PEX11β (Ebberink et al., 2012).

### Promoter Analysis of the *PEX11* Isoforms Suggests They Are Independently Regulated

**FIGURE 7:**
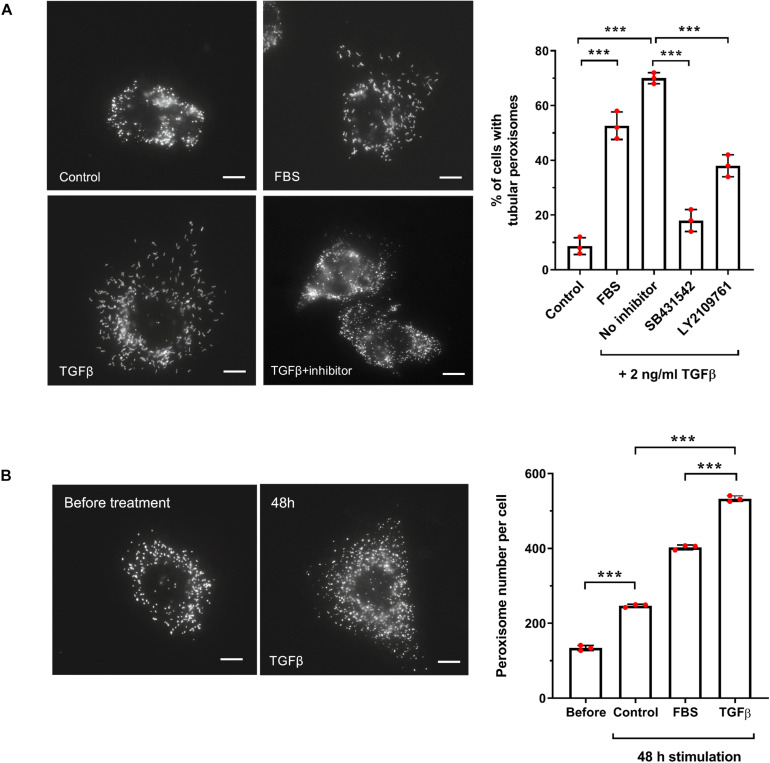
TGFβ increases peroxisome elongation and number in HepG2 cells. **(A)** HepG2 cells were cultured in MEM/N1, treated with 10% FBS, or TGFβ (2 ng/ml) (with or without inhibitors LY2109761 or SB431542) or mock treated and processed for immunofluorescence after 24 h after treatment using anti-PEX14 antibodies. Representative images and quantitative analysis of peroxisome morphology (elongation) for the conditions described are shown. Data from 3 independent experiments (*n* = 300 cells for each condition); analyzed by one-way ANOVA with Tukey’s *post hoc* test; ***p < 0.001. Bars, 10 μm. **(B)** HepG2 cells were cultured in MEM/N1 for 6 h prior to the addition of TGFβ (2 ng/ml) or 10% FBS, and processed for immunofluorescence after 6 h (‘before’ treatment) and 48 h after treatment as described in **(A)**. Representative images and quantitative analysis of peroxisome number/cell before and 48 h are shown. Data from 3 independent experiments (*n* = 10 cells for each condition); analyzed by one-way ANOVA with Tukey’s *post hoc* test; ***p < 0.001. Bars, 10 μm.

Since the mRNA profiles of the three *PEX11* isoforms differed during the peroxisome proliferation/division cycle, we set out to identify candidate factors leading to this differential regulation. A comparison of the promoter regions of all three human *PEX11* genes revealed that *PEX11*β and *PEX11*γ share four putative transcription factor binding sites for SMAD2/3 (Small worm phenotype/Mothers Against Decapentaplegic homolog 2/3), ATF4 (Activating Transcription Factor 4), SOX10 (Sex-determining Region Y-related High Mobility Group-box 10) and PPARγ. In contrast, no transcription factor binding sites are shared between *PEX11*β and *PEX11*α, while *PEX11*α and *PEX11*γ only share one binding site (for PPARα) (Figure 7). These findings indicate that *PEX11*β and *PEX11*α are likely to be differently regulated at a transcriptional level and further support our notion that both proteins fulfill different functions at peroxisomes.

### The Canonical TGFβ Pathway Is Involved in Peroxisome Proliferation in HepG2 Cells

One of the key activators of SMAD2/3 is transforming growth factor beta (TGFβ), via the canonical TGFβ signaling pathway. We chose to focus our subsequent studies on SMAD2/3-mediated regulation of *PEX11*β and the possible link between TGFβ signaling and peroxisome proliferation, as a proof-of-principle of our predictive promoter analysis. This was selected because of: (i) the functional role of PEX11β in peroxisome elongation and division (Figure 6); (ii) the high probability prediction of a SMAD2/3 binding site in the promoter region of *PEX11*β (Supplementary Table 6), and (iii) the ability of TGFβ to activate mTOR, a driver of peroxisome proliferation (Figure 4B), which together suggested that expression of PEX11β, and thus peroxisome proliferation, might be linked to TGFβ signaling. First, we tested whether TGFβ could induce peroxisome elongation and proliferation in our cell-based model. HepG2 cells cultured in MEM/N1 were mock treated or treated with 2 ng/ml recombinant TGFβ for 24 h prior to fixation and PEX14 immunofluorescence (Figure 2). Recombinant TGFβ activates Type I and, indirectly, Type II TGFβ receptors on the cell surface, resulting in phosphorylation of the SMAD2/3 complex and its translocation to the nucleus where it can modulate transcription of target genes (Dituri et al., 2013). Whereas control cells showed overwhelmingly spherical peroxisomes, addition of TGFβ resulted in a pronounced elongation of peroxisomes, a pre-requisite of peroxisomal growth and division (Figure 2A). To validate that the effect was specific to the SMAD-mediated TGFβ pathway, we used specific chemical inhibitors which block the TGFβ signaling pathway. SB431542 selectively inhibits Type I TGFβ receptors by suppressing SMAD3 phosphorylation, whereas LY2109761 inhibits both Type I and II receptors, resulting in reduced phosphorylation of SMAD2 (Dituri et al., 2013). Pre-treatment of HepG2 cells with either inhibitor for 2 h before adding recombinant TGFβ reduced peroxisome elongation significantly when compared to TGFβ-treated controls (Figure 2A) indicating a specific TGFβ-mediated response. Furthermore, consistent with the effect of serum stimulation, the number of peroxisomes in TGFβ-treated cells was significantly increased when compared to unstimulated controls (Figure 2B). 48 h TGFβ treatment resulted in an over two-fold increase in peroxisome numbers compared to untreated cells cultured for the same amount of time, which is indicative of accelerated peroxisome proliferation. Overall, these findings support a role for TGFβ signaling in peroxisome proliferation, potentially via SMAD2/3 binding to the *PEX11*β promoter.

**FIGURE 8:**
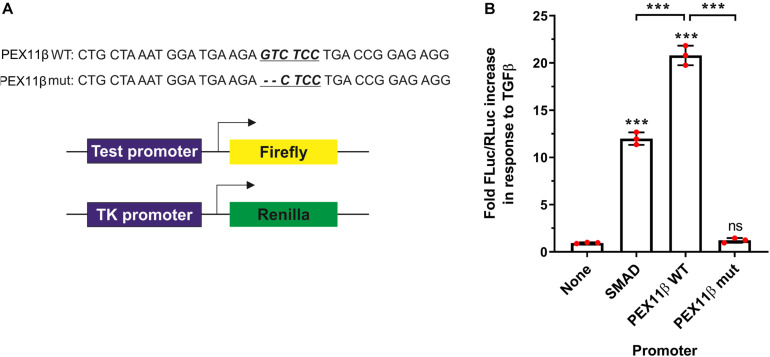
TGFβ activates *PEX11*β expression by direct binding of the SMAD2/3 transcription factor to the *PEX11*β promoter. Luciferase reporter assays in HepG2 cells transfected with a Firefly-Luc reporter under the control of a SMAD2/3 motif-containing promoter (‘SMAD’); or *PEX11*β wild type (‘WT’) or SMAD2/3 mutant (‘mut’) promoter. As a control, a promoter-less Firefly-Luc vector was used. Cells were treated with TGFβ (2 ng/ml) or left untreated. **(A)** Schematic of the reporter vectors used. The potential SMAD2/3 binding motif (underlined) in the *PEX11*β promoter and the deleted base pairs are indicated. pRL-TK construct with thymidine kinase promoter upstream of Renilla luciferase was used as an internal control in all the experiments. **(B)** Quantitative analysis of the effect of TGFβ on promoter activity. Firefly luciferase activity (FLuc) was first normalized to Renilla luciferase activity (RLuc) in each condition. Fold increase in response to TGFβ was then calculated by normalizing the relative luminometer unit values for each construct in the presence of TGFβ to the value without TGFβ. Data from 3 independent experiments; analyzed by one-way ANOVA with Tukey’s *post hoc* test; ns non-significant, ***p < 0.001.

To verify that transcription of *PEX11*β can be regulated by TGFβ in a SMAD-dependent manner, we transfected HepG2 cells with constructs consisting of a firefly luciferase reporter gene under the control of the *PEX11*β promoter, and assayed the effect of TGFβ on luminescence as a result of firefly luciferase expression (Figure 8). TGFβ treatment dramatically increased the expression of firefly luciferase driven by the wild type *PEX11*β promoter (or a control promoter with an optimized SMAD2/3 binding site, ‘SMAD’), relative to a constitutively expressed, TK promoter-driven Renilla luciferase as an internal control. However, mutating the putative SMAD2/3 binding site in the *PEX11*β promoter (‘mut’) prevented the TGFβ-dependent increase in reporter expression (Figure 8). These findings indicate that the SMAD2/3 binding site within the *PEX11*β promoter is functional, and that *PEX11*β transcription is positively regulated by TGFβ signaling via its SMAD2/3 binding site.

## Discussion

In this study, we used the well-differentiated human hepatoblastoma cell line HepG2 as a model system to assess the ability of different stimuli to induce peroxisome proliferation. The peroxisomal compartment in HepG2 cells is very dynamic, displaying high plasticity including the presence of elongated peroxisomes (Schrader et al., 1996; Grabenbauer et al., 2000). These elongated peroxisomes form by membrane expansion as a pre-requisite of peroxisome division and can be observed during rapid cellular growth, for example after hepatectomy (Yamamoto and Fahimi, 1987b) or in cultured mammalian cells (including COS-7 cells) (Schrader et al., 1996, 1998a; Duclos et al., 1997). Stimulation of cultured cells with defined growth factors, fatty acids or free radicals promotes peroxisome elongation (Schrader et al., 1998a, 1999) as does depolymerisation of microtubules (Schrader et al., 1996; Passmore et al., 2017), suggesting the involvement of intracellular signaling in peroxisome elongation. Motor-driven pulling forces, e.g., mediated by the kinesin-adaptor Miro1 along microtubules, can also contribute to peroxisomal membrane expansion (Castro et al., 2018) as well as inhibition of the peroxisomal division machinery (Koch et al., 2003; Gandre-Babbe and van der Bliek, 2008; Passmore et al., 2020) and tethering of peroxisomes to the ER (Costello et al., 2017).

We show here that peroxisome membrane expansion/elongation in HepG2 cells depends on serum/growth factors, and can be inhibited by rapamycin, an inhibitor of the mTOR pathway, the main growth factor-dependent pathway in mammalian cells (Wullschleger et al., 2006). Indeed, the mTOR pathway has recently been linked to peroxisome homeostasis and signaling (Zhang et al., 2013, 2015). Notably, the PPARγ antagonist GW9962 did not prevent the induction of peroxisome elongation by serum, while the PPARα agonist Wy-14,643 and the PPARγ ligand troglitazone only had a relatively small stimulatory effect on peroxisome elongation, and to a lower extent than serum addition. These observations are in line with earlier studies (Sher et al., 1993; Stangl et al., 1995; Duclos et al., 1997; Schrader et al., 1998b; Hsu et al., 2001; Lawrence et al., 2001; Bagattin et al., 2010) and point to the involvement of PPAR-independent pathways in peroxisome elongation/proliferation in humans. In a high-content screen probing more than 15,000 drugs, 10 compounds were reported to induce peroxisome proliferation in HepG2 cells, which appear to be only mildly sensitive to PPARα-mediated peroxisome proliferation (Sexton et al., 2010).

A stimulatory effect of PUFAs, especially AA [C20:4 (ω-6)] on peroxisomal membrane expansion confirms earlier studies (Schrader et al., 1998a). PA (C16:0), a preferential substrate for mitochondrial β-oxidation, was not effective in inducing elongation, supporting specificity for longer-chain/unsaturated fatty acids, which link to the synthesis of cellular lipid mediators. AA is the substrate for the synthesis of prostaglandins and leukotrienes. As peroxisomes are involved in the degradation of prostaglandins (Diczfalusy and Alexson, 1988; Schepers et al., 1988) and leukotrienes (Jedlitschky et al., 1991), peroxisome elongation/proliferation may help to balance intracellular levels of these eicosanoids. Furthermore, other PUFAs such as DHA [C22:6(*n*−3)] have previously been shown to mediate peroxisome elongation and division/proliferation (Itoyama et al., 2012). While PUFAs, including DHA, are well-characterized ligands of PPARα (Krey et al., 1997), DHA-induced peroxisomal proliferation has been shown to be independent of PPARα activation, but dependent on PEX11β and peroxisome division factors (Itoyama et al., 2012).

In line with this, *PEX11*β mRNA levels correlated well with peroxisome elongation and subsequent division/proliferation. *PEX14* mRNA levels were also slightly upregulated after 24 h in culture, when peroxisomes were also most elongated. Besides a role in matrix protein import, PEX14 has also been suggested to function as a microtubule docking factor stabilizing elongated peroxisomes, which may explain its induction during proliferation (Bharti et al., 2011; Theiss et al., 2012; Castro et al., 2018; Passmore et al., 2020). mRNA levels of the peroxisomal ABC transporter *ABCD3* (*PMP70*) correlated with the peroxisome proliferation cycle in line with previous studies (Schrader et al., 1998a). mRNA levels for the transcription factor *PPAR*α or *ACOX1*, a key enzyme in peroxisomal β-oxidation, did not correlate with the peroxisome proliferation cycle, which is perhaps surprising, as enzyme expression may be expected to rise in line with the increase in peroxisome number to maintain normal metabolism. However, mRNA and protein levels of *PPAR*α and branched-chain *ACOX2* have been previously shown to only be markedly elevated at the later phases of long-term culture and differentiation of HepG2 cells, suggesting that peroxisome proliferation (investigated here) and peroxisome maturation, characterized by a metabolically-active phenotype once cells reach confluency, may be two distinct and differentially regulated processes (Stier et al., 1998). *PGC1*α mRNA levels are also reported to increase after 3 days, and may link to an (differentiation-dependent) induction of peroxisomal activity (Bagattin et al., 2010; Huang et al., 2017).

We focussed on the function and regulation of the PEX11 proteins, which are supposed to be key regulators of peroxisome proliferation, although the functions of the different PEX11 isoforms in peroxisome proliferation are still controversial. They have been suggested to cooperate in the coordination of peroxisomal growth and division (Koch and Brocard, 2012), but functions in fatty acid oxidation, transport processes, organelle interplay and membrane contacts have also been reported (van Roermund et al., 2000; Dulermo et al., 2015; Mattiazzi Ušaj et al., 2015; Mindthoff et al., 2016; Kustatscher et al., 2019; Wu et al., 2020).

Our data reveal that PEX11β and PEX11α are likely to be differently regulated as different transcription factors are predicted to control their expression. Additionally, in contrast to *PEX11*β, *PEX11*α mRNA levels did not follow the changes in peroxisome morphology in HepG2 cells. Furthermore, expression of PEX11α did not induce peroxisome membrane expansion, which is consistent with previous studies (Schrader et al., 1998b; Delille et al., 2010). PEX11α cannot complement loss of PEX11β (Ebberink et al., 2012), and despite their similar membrane topology, both proteins show different sensitivity to Triton-X 100 pointing to different functions and biochemical properties within the peroxisomal membrane (Schrader et al., 2012a). Patients suffering from PEX11β deficiency have been identified (Ebberink et al., 2012; Taylor et al., 2017; Tian et al., 2019), whereas patients with a defect in PEX11α function have not yet been diagnosed. In line with this, knockout of PEX11β in mice is lethal, whereas PEX11α KO mice appear healthy (Li et al., 2002a, b). However, feeding of PEX11α KO mice with a high-fat diet resulted in impaired physical activity and energy expenditure, decreased fatty acid β-oxidation, increased *de novo* lipogenesis as well as dyslipidaemia and obesity (Chen et al., 2019). Overall, these findings highlight that PEX11α and PEX11β have different functions, and may support a role for PEX11α in peroxisomal fatty acid metabolism rather than in peroxisome proliferation.

We confirmed that the identified putative SMAD2/3 binding site in the promoter region of PEX11β is functional and TGFβ-responsive, and could potentially link peroxisome proliferation to the canonical, SMAD-dependent TGFβ signaling pathway in HepG2 cells. A stimulatory effect of TGFβ on peroxisome elongation, albeit less prominent due to altered experimental conditions, was observed in a previous study (Schrader et al., 1998a). Other genes containing putative SMAD2/3 binding sites include *PEX11*γ, *FIS1*, *PEX13* and *PEX14* (Supplementary Table 6) further indicating the potential involvement of this pathway in the regulation of genes involved in peroxisome dynamics, and these genes may therefore also contribute to the TGFβ-induced peroxisome proliferation, along with *PEX11*β. Previous work has demonstrated a reduction in PEX13 and PEX14 expression in fibroblasts following TGFβ stimulation (Oruqaj et al., 2015), which is perhaps surprising given our data suggests TGFβ promotes peroxisome proliferation, and we find *PEX13* mRNA stays the same, while *PEX14* mRNA increases, during FBS-induced peroxisome proliferation (Figure 3). This could be a result of tissue-specific differences in the TGFβ-dependent regulation of peroxisome proliferation, or differences in the regulation of *PEX13* and *PEX14* by serum factors as opposed/in addition to TGFβ.

The TGFβ pathway is involved in the regulation of cellular proliferation, differentiation, embryogenesis, apoptosis, inflammation, immunity and cancer pathways (Massagué, 2012). TGFβ is also a key regulator of liver physiology and pathology. It contributes to hepatocyte proliferation and differentiation, but also to all stages of disease progression, from initial liver injury through inflammation and fibrosis to cirrhosis and hepatocellular carcinoma (Fabregat et al., 2016). HepG2 cells have been shown to express TGFβ and to respond to TGFβ treatment or silencing of TGFβ with alterations in cell growth, apoptosis and the cell cycle (Wang et al., 2017). We assume that TGFβ signaling in our experimental system contributes to the proliferation and/or differentiation of HepG2 cells as peroxisome elongation is a hallmark of cell growth and proliferation (Schrader et al., 1999). Interestingly, elongated and tubular or constricted peroxisomes were also observed after partial hepatectomy in rat liver (Yamamoto and Fahimi, 1987a). These heterogenous peroxisome morphologies were interpreted as an indicator of peroxisome proliferation by elongation/growth and division under conditions of cellular growth and proliferation (Schrader and Fahimi, 2008). TGFβ has a crucial role in hepatocytes *in vivo*, being critical for the control of liver mass (Karkampouna et al., 2012). The TGFβ pathway has activator and repressor effects on other pathways such as cell growth, so balanced TGFβ signaling in terms of both dosage and spatiotemporal activity is crucial to control hepatic gene expression. TGFβ acts in the process of differentiation of hepatoblasts to hepatocytes during liver regeneration after partial hepatectomy (Karkampouna et al., 2012). The TGFβ pathway is required at different stages of the process, to allow hepatocyte proliferation at the inductive phase followed by an efficient termination of the regenerative response afterward.

Since HepG2 cells are hepatoblastoma cells, which can differentiate in culture and form bile canaliculi-like structures (Bokhari et al., 2007), we propose that, similar to regenerating liver, TGFβ induces peroxisome proliferation in HepG2 cells under proliferative culture conditions, possibly via upregulation of *PEX11*β, a key regulator of peroxisomal growth and multiplication. Under standard culture conditions, peroxisome elongation has a maximum after 24–48 h, before the elongated peroxisomes divide resulting in the formation of numerous spherical peroxisomes. Peroxisomes do not massively elongate at later time points in culture, when the cells are more confluent, and therefore growing more slowly (Schrader et al., 1998a). Ultrastructural studies have shown that at prolonged culture the peroxisomes in HepG2 cells are larger and form small clusters of organelles, possibly changing from a proliferative to a metabolic state (Grabenbauer et al., 2000). As PEX11β mediates peroxisome elongation and proliferation, it represents an ideal target to adapt peroxisome number and function according to cellular needs. TGFβ signaling may foster peroxisome proliferation in an inductive phase of HepG2/hepatocyte proliferation and differentiation, but may also reduce proliferation of peroxisomes afterward, when the inductive stage is completed, and cell growth needs to be terminated. As TGFβ signaling is complex, it is likely that other, non-canonical signaling pathways as well as TGFβ secretion, the concentration of active TGFβ, and the amount of TGFβ receptors and spatiotemporal activity, contribute to the overall cellular response. Additionally, PEX11β expression is likely to be subject to other levels of regulation, such as translational control or alterations in protein stability, localization or activity, that coordinate to fine-tune peroxisome proliferation in response to various conditions, which is an interesting avenue for future investigation.

## Conclusion

We reveal a new TGFβ-dependent signaling pathway controlling peroxisome proliferation which may act by regulating *PEX11*β gene expression. This opens a possibility for therapeutic approaches controlling regulation of *PEX11*β in patients where peroxisome abundance/activity is reduced.

## Data Availability Statement

All datasets generated for this study are included in the article/Supplementary Material.

## Author Contributions

AA, RC, WK, JK, and SK performed experiments and analyzed the data. MS, WK, and HW conceived the project and analyzed data. MS and RC wrote the manuscript. All authors contributed to methods.

## Conflict of Interest

The authors declare that the research was conducted in the absence of any commercial or financial relationships that could be construed as a potential conflict of interest.

## Footnotes

1 http://crispr.mit.edu/

2 http://jaspar.genereg.net/

## Supplementary Material

Supplementary Figure 1 Cellular growth curve based on cell number at the indicated time points. An exponential increase in cell density is observed in both cells cultured in MEM/N1 and MEM/FBS. Data are expressed as mean total cell number (per well) from 3 independent experiments. Measurements were performed in triplicate.

Supplementary Figure 2 Effect of PPAR agonists and antagonists on peroxisome morphology in HepG2 cells. Quantitative analysis of peroxisome morphology in HepG2 cells cultured in MEM/N1 after treatment with the PPARα agonist Wy-14,643 (10–20 μM) **(A)**, the PPARγ agonist Troglitazone (5–15 μM) **(B)**, or pre-treatment with the PPARγ antagonist GW9962 (20 μM) prior to serum stimulation **(C)**. Treatment with FBS served as a positive control. Note that the PPARγ agonist Troglitazone and the PPARα agonist Wy-14,643 induce peroxisome elongation to a lesser extent than FBS. Furthermore, the PPARγ antagonist GW9962 does not repress serum-induced peroxisome elongation/proliferation. Data are based on immunofluorescence microscopy performed 24 h after treatment using anti-PEX14, from 3 independent experiments (*n* = 300 cells in each condition); analyzed by one-way ANOVA **(A,B)** or two-way ANOVA **(C)** with Tukey’s *post hoc* test; ***p* < 0.01, ****p* < 0.001.

Supplementary Table 1 Details of plasmids used in this study.

Supplementary Table 2 Plasmids generated in this study.

Supplementary Table 3 Human qPCR primers used in this study.

Supplementary Table 4 Primary and secondary antibodies used in this study.

Supplementary Table 5 Generation of PEX11-deficient HeLa cells.

Supplementary Table 6 Predicted SMAD2/3 binding sites in peroxisomal gene promoters. Start and end position expressed in relation to 10 kb region upstream of the transcription start site. Binding efficiency calculated from the SMAD2/3 JASPAR position-weight matrix consensus sequence (ID MA0513.1).

## Abbreviations

AA: arachidonic acid
ABCD: ATP-binding cassette sub-family D
ACAA1: acetyl-CoA acyltransferase 1
ACOX1: acyl-CoA oxidase 1
AGPS: alkylglycerone phosphate synthase
CRAT: carnitine *O*-acetyltransferase
CROT: carnitine *O*-octanoyltransferase
DHA: docosahexaenoic acid
DRP1: dynamin-related protein 1
EHHADH: enoyl-CoA hydratase and 3-hydroxyacyl-CoA dehydrogenase
FAR1: fatty acyl-CoA reductase 1
FBS: fetal bovine serum
FIS1: mitochondrial fission 1 protein
GNPAT: glyceronephosphate *O*-acyltransferase
LA: linoleic acid
MFF: mitochondrial fission factor
mTOR: mammalian target of rapamycin
OA: oleic acid
PA: palmitic acid
PEX: peroxisome biogenesis factor (peroxin)
PGC1: PPAR gamma coactivator 1
PMP: peroxisomal membrane protein
PPAR: peroxisome proliferator activated receptor
PPRE: peroxisome proliferator response element
PUFA: polyunsaturated fatty acid
SMAD: small worm phenotype/mothers against decapentaplegic homolog
TGF β: transforming growth factor β
VLCFA: very-long-chain fatty acid.
