# Supplementary figures and images for: A Functional SMAD2/3 Binding Site in the PEX11β Promoter Identifies a Role for TGFβ in Peroxisome Proliferation in Humans
Source: Front Cell Dev Biol. 2020 Oct 23;8:577637. doi: 10.3389/fcell.2020.577637 (PMC7644849; doi:10.3389/fcell.2020.577637)

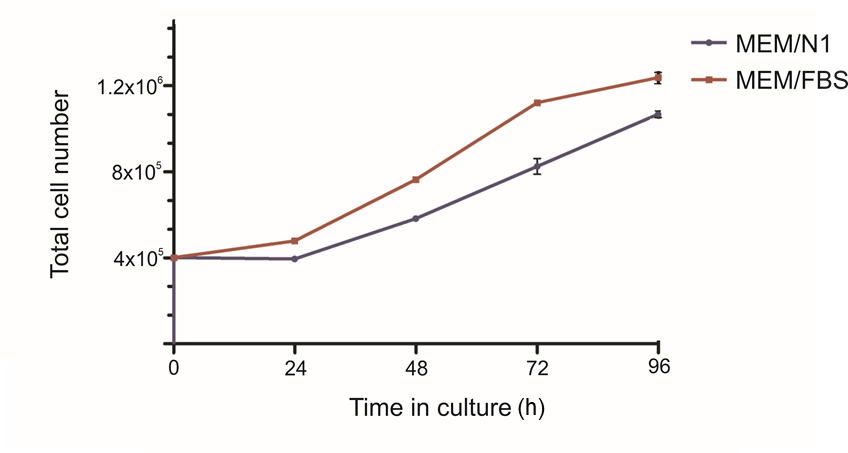

Supplement: Supplementary Figure 1 — Cellular growth curve based on cell number at the indicated time points. An exponential increase in cell density is observed in both cells cultured in MEM/N1 and MEM/FBS. Data are expressed as mean total cell number (per well) from 3 independent experiments. Measurements were performed in triplicate. [file Image_1.TIF]

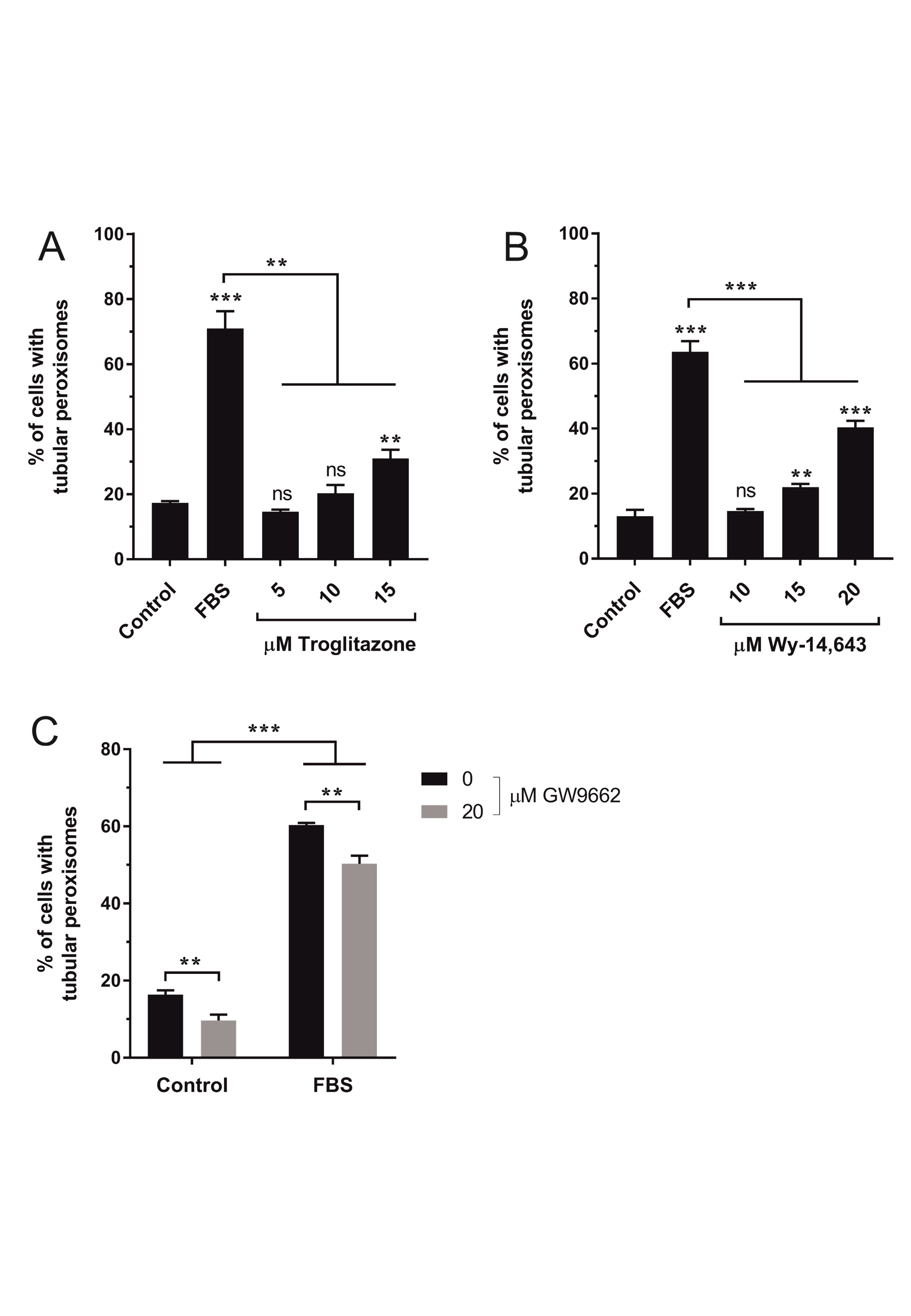

Supplement: Supplementary Figure 2 — Effect of PPAR agonists and antagonists on peroxisome morphology in HepG2 cells. Quantitative analysis of peroxisome morphology in HepG2 cells cultured in MEM/N1 after treatment with the PPARα agonist Wy-14,643 (10–20 μM) (A), the PPARγ agonist Troglitazone (5–15 μM) (B), or pre-treatment with the PPARγ antagonist GW9962 (20 μM) prior to serum stimulation (C). Treatment with FBS served as a positive control. Note that the PPARγ agonist Troglitazone and the PPARα agonist Wy-14,643 induce peroxisome elongation to a lesser extent than FBS. Furthermore, the PPARγ antagonist GW9962 does not repress serum-induced peroxisome elongation/proliferation. Data are based on immunofluorescence microscopy performed 24 h after treatment using anti-PEX14, from 3 independent experiments (n = 300 cells in each condition); analyzed by one-way ANOVA (A,B) or two-way ANOVA (C) with Tukey’s post hoc test; **p < 0.01, ***p < 0.001. [file Image_2.TIF]
