# Supplementary material for: A Functional SMAD2/3 Binding Site in the PEX11β Promoter Identifies a Role for TGFβ in Peroxisome Proliferation in Humans
Source: Front Cell Dev Biol. 2020 Oct 23;8:577637. doi: 10.3389/fcell.2020.577637 (PMC7644849; doi:10.3389/fcell.2020.577637)
Supplement: Supplementary Table 1 — Details of plasmids used in this study. [file Data_Sheet_1.docx]

Table S1. **Plasmids used in this study**

| **Plasmid** | **Source** |
| --- | --- |
| Myc-PEX11α | Delille *et al.*, 2010 |
| Myc-PEX11β | Delille *et al.*, 2010 |
| Myc-PEX11γ | Schrader *et al.*, 2012 |
| pGL3-basic | Promega #E1751 |
| pRL-TK | Promega #E2231 |

Table S2. **Plasmids generated in this study**

| **Name** | **Insert (± SMAD binding site - underlined)** | **Enzymes** | **Vector** |
| --- | --- | --- | --- |
| *PEX11β* WT promoter | CCCCAAGCGCACCCTTTAACTCATGCTATGGACCCAAATCCCTGTCCTGCCCGGTCCTGAGTCCTAGGTCACTCACCTGGGGGAAGGCAAGCAGGAAACTAAGTCCCCAGGCTGCCCCCAGAAGTTTCCTTACACCTGAACGGGATCCAAGCGGGTTGAGTACTGCTGCCTGGCGGTCCAATCCAATGACCACAGGCAGGAAAGCTGCAGAATACGTGGCCATTAGTTTCAGGAACATCAGTGTCCGACATGCGATGTCCACAGCCAGCCATTGAACAGTGATATTCCAGGTGGCATCTAGGGGCATAACCACAAAAGTGACTAGTAAGTCGGCGGCTGCTAAATGGATGAAGA**GTCTCC**TGACCGGAGAGGGGCGGAGCTGGCTGGGTTCCCGCCGTGTCACTGACCACAGGACTGCCAGGTTCCCTCCAGCCGAAGAAACAAACAGCACAATGGTCACTCCCACTCGGACCTTGGCTGCTGCCGAGAAGGTGGGCAGCTCTGAGCCCTCCACCTCCACTCCTGATCCAGCCCAGACCTCCTCCCCCGCTGCTGACCCCAAGGGGTGCCGTTGCCTGCAGACATGGTGGCCTGGAGAGAAACTGAGGAGGATGAAGTGTGGGTATGAAGCGGTTAGCTTTGGCCACTGATTCTTCGCCCTCCTTTTCAGGATCTGGAGCTGATTATTGCGAATTTCGTTCACTAAAGCCTCTGCTTCTGGAGACTCTCCATCTGTTCTGGAGGAGGTCACTTGTTCAACTGTCAAGGTTCTGCAGGGAACGAAGATCTGGTACCAATCACGCTCCTTTTTCCTTGGACAGACCAGCCTGGGCTTCAGGCCTCTACTGACCTAGACCTCTTTAGCCCCTACAGCCCTGGATTCAGTCGGGGCCTGCGTGCATGTGTATTTGTGAGGTTCTGCCAGGCGCACACCTATGCCGGCGGCCTCCAAGACGTGGAGATCACTGCGGCGCTTTCTCGCCCCGCCCCGGCCCCTGCAGGCCCCGCCCTTGGTCATGAATATTTAAAGAAGAAGGTGCCGCTGGAGGCGTGCTAGGGAGTAGGGGTCGTCTGATAAGGGGAAGCTGTGACGCAGACACGCACAGTAATACACAGATGGAGGCTCAAAAGACACGAGTTTCGCGTCCTGAAATTCCGCTTCCAGGGCCAAGCTTTCTTTTCTGATACTGTTTGTCCCTCGCGAGGCACCGTTGGGTCGCGCAGTAGGCGTGACTAGGGGCGGGAAGTGGGGCGGGAGCAGGGCCGCGGAGCCTGGGCTGCGGCTGTCAT | SacI  XhoI | pGL3-basic |
| *PEX11β* mut promoter | CCCCAAGCGCACCCTTTAACTCATGCTATGGACCCAAATCCCTGTCCTGCCCGGTCCTGAGTCCTAGGTCACTCACCTGGGGGAAGGCAAGCAGGAAACTAAGTCCCCAGGCTGCCCCCAGAAGTTTCCTTACACCTGAACGGGATCCAAGCGGGTTGAGTACTGCTGCCTGGCGGTCCAATCCAATGACCACAGGCAGGAAAGCTGCAGAATACGTGGCCATTAGTTTCAGGAACATCAGTGTCCGACATGCGATGTCCACAGCCAGCCATTGAACAGTGATATTCCAGGTGGCATCTAGGGGCATAACCACAAAAGTGACTAGTAAGTCGGCGGCTGCTAAATGGATGAAGA**********TGACCGGAGAGGGGCGGAGCTGGCTGGGTTCCCGCCGTGTCACTGACCACAGGACTGCCAGGTTCCCTCCAGCCGAAGAAACAAACAGCACAATGGTCACTCCCACTCGGACCTTGGCTGCTGCCGAGAAGGTGGGCAGCTCTGAGCCCTCCACCTCCACTCCTGATCCAGCCCAGACCTCCTCCCCCGCTGCTGACCCCAAGGGGTGCCGTTGCCTGCAGACATGGTGGCCTGGAGAGAAACTGAGGAGGATGAAGTGTGGGTATGAAGCGGTTAGCTTTGGCCACTGATTCTTCGCCCTCCTTTTCAGGATCTGGAGCTGATTATTGCGAATTTCGTTCACTAAAGCCTCTGCTTCTGGAGACTCTCCATCTGTTCTGGAGGAGGTCACTTGTTCAACTGTCAAGGTTCTGCAGGGAACGAAGATCTGGTACCAATCACGCTCCTTTTTCCTTGGACAGACCAGCCTGGGCTTCAGGCCTCTACTGACCTAGACCTCTTTAGCCCCTACAGCCCTGGATTCAGTCGGGGCCTGCGTGCATGTGTATTTGTGAGGTTCTGCCAGGCGCACACCTATGCCGGCGGCCTCCAAGACGTGGAGATCACTGCGGCGCTTTCTCGCCCCGCCCCGGCCCCTGCAGGCCCCGCCCTTGGTCATGAATATTTAAAGAAGAAGGTGCCGCTGGAGGCGTGCTAGGGAGTAGGGGTCGTCTGATAAGGGGAAGCTGTGACGCAGACACGCACAGTAATACACAGATGGAGGCTCAAAAGACACGAGTTTCGCGTCCTGAAATTCCGCTTCCAGGGCCAAGCTTTCTTTTCTGATACTGTTTGTCCCTCGCGAGGCACCGTTGGGTCGCGCAGTAGGCGTGACTAGGGGCGGGAAGTGGGGCGGGAGCAGGGCCGCGGAGCCTGGGCTGCGGCTGTCAT | SacI  XhoI | pGL3-basic |

Table S3. **Human** **qPCR primers used in this study**

| **Gene name** | **Forward primer sequence**  **(5’ to 3’)** | **Reverse primer sequence**  **(5’ to 3’)** | | **Amplicon length (bp)** |
| --- | --- | --- | --- | --- |
| *18S rRNA* | GTAACCCGTTGAACCCCATT | | CCATCCAATCGGTAGTAGCG | 151 |
| *PEX11α* | GAGAAGGTGGTAATGAAGCTCAA | | GCTCTGCTCAGTTGCCTGT | 108 |
| *PEX11β* | CGCCCAGTATGCTTGCTCTC | | TCGATTGAGGTGACTAACAGTGA | 220 |
| *PEX11γ* | GGGGACACGTCTGTTGGTG | | ACAGGGGTAGTAGAGCTGGTC | 184 |
| *PEX1* | GGGGACAGGTATTTCTCAAGC | | CCAGCCTTCCATAAGAGGCAG | 243 |
| *PEX2* | GAGAATGCGAAGAGTGCAAACA | | CTGGCTCAAAGCGAGCTAACA | 148 |
| *PEX3* | GGCTGAGTTCTTTCGACCTA | | ACTGCAAACTGAATGGATCTGTC | 131 |
| *PEX5* | AAGCCTTTGGGAGTAGCTTCT | | GGACACAAGGGGTGCATTC | 75 |
| *PEX6* | CCCTTTCCGACCGAGACAC | | CGCGGCTAACCAGTAGCTG | 205 |
| *PEX10* | TCTGCTGGGAGTGCATCAC | | CGAAGGTAGATGAGCTTCTGGG | 94 |
| *PEX12* | TTGAGGTGGTAGCACAGGACA | | GTGGGATTTGATTCTGCAAGAAC | 88 |
| *PEX13* | ACCTGGACAACCAGCACTTAC | | GCCCAGCCCATTATATCCATAAC | 187 |
| *PEX14* | GCCACGGCAGTGAAGTTTCTA | | TGCTGGAAGGCCATATCAATCT | 119 |
| *PEX16* | GTGCGGGGCTTCAGTTACC | | GGTTAGAGGCAGAGTACACCA | 82 |
| *PEX19* | GCTGAGGAAGGCTGTAGTGTC | | CTGGCGATCTCTTCTGGGG | 157 |
| *PEX26* | GTGCTCCCTGTGTGTTGTG | | GGACCTGGTAATACTGAAGGAC | 93 |
| *PPARα* | ACACTGTGTATGGCTGAGAAGA | | GACGGTCTCCACTGACGTG | 117 |
| *PPARγ1* | AAAGAAGCCAACACTAAACC | | CTTCCATTACGGAGAGATCC | 150 |
| *PGC1α* | TCTGAGTCTGTATGGAGTGACAT | | CCAAGTCGTTCACATCTAGTTCA | 112 |
| *PGC1β* | GATGCCAGCGACTTTGACTC | | ACCCACGTCATCTTCAGGGA | 186 |
| *ABCD1* | GCTGGCATGAACCGGGTATT | | GCCACATACACCGACAGGAA | 143 |
| *ABCD3* | GCCTGCACGGTAAGAAAAGTG | | AGCCTTGAGAAAAACACCTTGTC | 100 |
| *ACOX1a* | CCTGAACGACCCAGACTTCC | | TGCCTGGTGAAGCAAGGTG | 227 |
| *ACOX1b* | GGGCCTCAATTACTCCATGTTT | | TGGGCGTAGGTGCCAATTATC | 114 |
| *ACAA1* | GCGGTTCTCAAGGACGTGAAT | | GTCTCCGGGATGTCACTCAGA | 128 |
| *EHHADH* | GGTCAACGCGATCAGTACGAC | | CCTCTGCTCCACAAATCACAATG | 104 |
| *AGPS* | TTAGTGGCATGGGTTTACCAAC | | CTCGATCATCTGCCTCTTGTG | 189 |
| *GNPAT* | GAGGAGGCATGTCAGTGACTT | | ACAAAACCGAATGGCTCCAAG | 244 |
| *FAR1* | AGACACCACAAGAGCGAGTG | | CCAGTTTAGGTTGGGTGAGTTC | 126 |
| *CROT* | GTGGTGGCTGAATGTTGCCTA | | TTGGAGGCCAGTAGTGTTCAA | 101 |
| *CRAT* | GTGGCTCAAGACCGCCTAC | | GCAGCAAATCGGAGCTGAC | 117 |
| *PMP34* | CTTCGACTTCAGGTTGATGAGA | | TGACCTTTGACCCAGAGTGC | 188 |

Table S4. **Primary and secondary antibodies used in this study**

| **Antibodies** | **Type** | **Dilution (IMF)** | **Source** |  |
| --- | --- | --- | --- | --- |
| Myc (9E10) | mc ms | 1:200 | Santa Cruz Biotechnology (sc40) | |
| PEX14 | pc rb | 1:1400 | D.Crane, Griffith University, Australia  (Nguyen *et al.*, 2006; Grant *et al.*, 2013) | |
| AlexaFluor 488 IgG | dk anti-rb | 1:500 | Invitrogen (A21206) | |
| AlexaFluor 594 IgG | dk anti-ms | 1:1000 | Invitrogen (A21203) | |

Abbreviations: IMF, immunofluorescence; mc, monoclonal; pc, polyclonal; ms, mouse; rb, rabbit; dk, donkey

Table S5. **Generation of PEX11-deficient HeLa cells**

|  | **gRNA sequence** | **Mutation** | |
| --- | --- | --- | --- |
| *PEX11α* | TTTACGACCAGTGCTCACACTGG | | c. 103insA/101_104delCCAA + 103insA |
| *PEX11β* | CAAGAAGAGAGCAAGCATACTGG | | c. 70insT/70_73GCTT>TAGG |
| *PEX11γ* | ACGTGGACTCTTCTCGGTGGTGG | | c. 373delT/373delT |

Table S6. **Predicted SMAD2/3 binding sites in peroxisomal gene promoters.** Start and end position expressed in relation to 10 kb region upstream of the transcription start site. Binding efficiency calculated from the SMAD2/3 JASPAR position-weight matrix consensus sequence (ID MA0513.1).

| **Gene name** | **Start position** | **End position** | **Predicted SMAD binding site** | **Binding efficiency** |
| --- | --- | --- | --- | --- |
| *PEX11β* | 4339 | 4351 | TGAAGAGTCTCCTGA | 14.129957 |
| *PEX11γ* | 2003 | 2015 | CAGGCTCTCACCT | 9.831968053 |
| *FIS1* | 947 | 959 | GTGGCTCACACCT | 12.7058425 |
| *PEX13* | 5191 | 5203 | CTGTCTGTCTCAT | 10.062138 |
| *PEX14* | 6743 | 6755 | ATGTCTGGCACAT | 12.14857929 |

Delille, H. K., Agricola, B., Guimaraes, S. C., Borta, H., Lüers, G. H., Fransen, M., et al. (2010). Pex11pbeta-mediated growth and division of mammalian peroxisomes follows a maturation pathway. J. Cell Sci. 123, 2750–2762. doi:10.1242/jcs.062109.

Grant, P., Ahlemeyer, B., Karnati, S., Berg, T., Stelzig, I., Nenicu, A., et al. (2013). The biogenesis protein PEX14 is an optimal marker for the identification and localization of peroxisomes in different cell types, tissues, and species in morphological studies. Histochem. Cell Biol. 140, 423–442. doi:10.1007/s00418-013-1133-6.

Nguyen, T., Bjorkman, J., Paton, B. C., and Crane, D. I. (2006). Failure of microtubule-mediated peroxisome division and trafficking in disorders with reduced peroxisome abundance. J. Cell Sci. 119, 636–645. doi:10.1242/jcs.02776.

Schrader, M., Almeida, M., and Grille, S. (2012). Postfixation detergent treatment liberates the membrane modelling protein Pex11beta from peroxisomal membranes. Histochem. Cell Biol. 138, 541–547.
